# Assessment of Growth Reduction of Five Clinical Pathogens by Injectable S53P4 Bioactive Glass Material Formulations

**DOI:** 10.3389/fbioe.2020.00634

**Published:** 2020-06-26

**Authors:** Eline G. J. Thijssen, Nicole A. P. van Gestel, Raymond Bevers, Sandra Hofmann, Jan Geurts, Inge H. M. van Loo, J. J. Arts

**Affiliations:** ^1^Laboratory for Experimental Orthopedics, Department of Orthopedic Surgery, Research School CAPHRI, Maastricht University Medical Centre, Maastricht, Netherlands; ^2^Orthopaedic Biomechanics, Department of Biomedical Engineering, Eindhoven University of Technology, Eindhoven, Netherlands; ^3^Institute for Complex Molecular Systems, Eindhoven University of Technology, Eindhoven, Netherlands; ^4^Department of Medical Microbiology, Research School CAPHRI, Maastricht University Medical Centre, Maastricht, Netherlands

**Keywords:** biomaterials, S53P4, bioglass, eradication, S53P4 formulations, bacteria

## Abstract

The one-stage treatment of chronic osteomyelitis with S53P4 bioactive glass (BAG) granules has shown excellent results. However, these granules possess suboptimal handling properties. Therefore, new injectable S53P4 putty materials have been developed by the incorporation of a synthetic binder to contain glass granules. The goal of the current study was to assess their potential to eradicate five clinically relevant pathogens: *methicillin sensitive Staphylococcus aureus* (MSSA), *methicillin resistant Staphylococcus aureus* (MRSA), *Enterococcus coli* (*E. coli*), *Enterococcus faecalis* (*E. faecalis*), and *Pseudomonas aeruginosa* (*P. aeruginosa*). As a control, S53P4 granules (500–800 μm) and S66 glass (< 45 μm) were used. To evaluate the antimicrobial properties, the materials were cultured with the pathogens in a Müller-Hinton II broth for a week with daily colony forming unit (CFU) counting. One of the tested putty formulations was observed to reduce the number of CFU/mL compared to a negative control (no material, only pathogen in broth) for *E. coli, E. faecalis* and *P. aeruginosa*. However, none of the tested putty formulations was able to completely eradicate the pathogens in the broths, which would be needed for safe infection treatment. The results obtained for the control materials were unexpected. S66 glass showed full eradication of *P. aeruginosa* and reduced the number of CFUs of other pathogens, while the S53P4 granules did not show eradication. The observations on the loose S53P4 granules in this study contradict available literature, which needs further investigation. The results obtained in this study also stretch the importance for a better understanding of the underlying antimicrobial mechanism of S53P4 BAG and how this is related to the dosage. In addition, it should be elucidated how these antimicrobial properties are affected by changes in the material formulation, for example by addition of binders to improve the handling properties or by changing the surface area.

## Introduction

Chronic bone infections, or chronic osteomyelitis, are a major problem in the field of orthopedic surgery. Invasive treatment is needed to prevent the loss of the affected limb, sepsis or even death (Parsons and Strauss, 2004). For years the gold standard treatment consisted of a two-stage surgical treatment. During the first surgery, an excessive debridement of the infected tissues is performed, followed by the implantation of a local antibiotic carrier (e.g., poly- (methyl methacrylate) (PMMA) beads loaded with gentamycin). When the infection is completely eradicated, the antibiotic carrier is removed, and the bone defect is grafted with either autograft or allograft bone for reconstruction in a second surgery. In addition to the surgical treatment, systemic antibiotics, specific for the cultured strains, are administered for at least 6 weeks (2 weeks intravenously and 4 weeks orally) (Walenkamp et al., 1998; Geurts et al., 2016; Lindfors et al., 2016).

Osteomyelitis causes vasoconstriction of local vessels, diminished vessel quality and poor penetration of systemic antibiotics into the bone (Calhoun et al., 2009). Therefore, systemic antibiotics alone may not achieve sufficiently high doses at the site of infection and a localized administration of antibiotics is needed in order to treat the osteomyelitis. Another challenging factor in the treatment of chronic osteomyelitis is the worldwide increase of antibiotic-resistant bacteria (Geurts et al., 2016; Vugt et al., 2016). Therefore, it is important to develop biomaterials that have a different mechanism to eradicate bacteria, compared to current antibiotics (Drago et al., 2015). S53P4 bioactive glass (Bonalive® Biomaterials Ltd., Turku, Finland) has shown to be clinically effective in the treatment of chronic osteomyelitis (McAndrew et al., 2013; Geurts et al., 2016; Lindfors et al., 2016). The S53P4 bioactive glass is believed to increase the local pH and osmotic pressure through an exchange of ions with the environmental fluid, which is believed to result in bacterial death. In addition, the use of this biomaterial has been reported to be (cost) effective in the treatment of osteomyelitis, in a one-stage surgical procedure when accompanied by systemic antibiotic administration (Lindfors et al., 2016).

Currently, the major drawback of loose S53P4 BAG granules is the handling. Surgeons prefer an injectable and moldable biomaterial. To accommodate this, novel S53P4 bioactive glass putty formulations have been developed (van Gestel et al., 2017). These formulations consist of S53P4 bioactive glass granules surrounded by a synthetic binder of poly(ethylene) glycol (PEG) and glycerol. Currently, the putty formulations are used as a filler of bone defects. However, it remains unclear whether and how the incorporation of the binder would affect the antibacterial properties of the S53P4 granules. Therefore, the goal of the current study was to assess the antimicrobial properties of the newly developed injectable putty formulations against 5 clinically relevant bacteria strains. It was investigated whether these biomaterials could reduce the number of colonies over time compared to S53P4 granules and a S66 glass control group.

## Materials and Methods

### Pathogens

This study was performed to determine the antimicrobial activity of four biomaterials on five different bacterial strains: *methicillin sensitive Staphylococcus aureus (MSSA; ATCC 29213), methicillin resistant Staphylococcus aureus (MRSA; ATCC 12493), Enterococcus coli (ATCC 25922), Enterococcus faecalis (ATCC 29212)*, and *Pseudomonas aeruginosa (ATCC 27853)*. Per strain, a clean colony was entered in 5 mL sterile Müller-Hinton II broth (MH-II broth, Merck KGaA, Darmstadt, Germany) and cultured overnight at 37°C and 5% CO_2_ (Balouiri et al., 2016). The bacterial cultures were diluted with sterile MH-II broth until 0.5 McFarland (1.5 × 10^8^ CFU/mL) using a McFarland measure (Grant Bio Densitometer DEN-1, Grant Instruments^TM^, Cambridge, Great Britain). These solutions were then further diluted to approximately 1.5 × 10^7^ CFU/mL (experimental pathogen solution).

### Biomaterials

The biomaterials included S53P4 bioactive glass (500–800 μm granules), Putty A, Putty B (Bonalive® Biomaterials Ltd.) and loose S66 powder (Table 1). The biomaterials were added to 2 mL fresh MH-II broth (without pathogens) and incubated overnight (16–18 h) on a rolling plate (IKA® roller 6 digital, IKA®-Werke GmbH & Co. KG, Staufen, Germany) at room temperature. This overnight incubation was performed to precondition the broths by the ions released from the different biomaterials. Per tested biomaterial, 11 test tubes were preconditioned of which 10 were used to for the pathogen cultures (*n* = 2 per bacterial strain) and 1 was used as a negative control to evaluate the pH of the broth with biomaterial, over time. The pH was measured with a Litmus red paper and a pH meter (FiveEasy F20, Mettler Toledo®, Tiel, The Netherlands); no bacteria were added to this specific test tube.

**Table 1:** Experimental groups and corresponding concentrations of the tested biomaterials in the MH-II broth.

**Biomaterial**	**Glass compositions and biomaterial formulations [wt%]**	**Concentration [mg biomaterial/mL]**
S53P4 granules (500–800 μm)	53% SiO, 2.4% P_2_O_5_, 23% Na_2_O, 20% CaO	400
Putty A	37% binder, 63% S53P4^*^	645.8
Putty B	22% binder, 78% S53P4^*^	670.9
S66 powder (<45 μm)	66.4% SiO_2_, 9.5% Na_2_O, 3.3% CaO, 6.1% Ba, 6.3% K_2_O, 2.2% Cr, 4.6% Pb	400.3

* *The binder consists of polyethylene glycols (PEGs), glycerol, S53P4 granules of 500–800 μm, and S53P4 <45 μm powder. The specific compositions are confidential (Bonalive® Biomaterials Ltd.)*.

### Pathogen Cultures With Biomaterials

After the overnight incubation (T0), the broths with biomaterials were tested for their capacity to reduce the number of colony forming units (CFUs). 2 mL of the experimental pathogen solution was added per tube (*n* = 4 per bacterial strain for every biomaterial). Per culture, one growth or positive control was added, which was a test tube with fresh MH-II broth, without biomaterials, but with pathogens (*n* = 4). All test tubes were then cultured for 7 days at 37°C and 5% CO_2_. During this 7-day culture, the colonies were counted daily.

### Colony Counting

**Figure 1:**
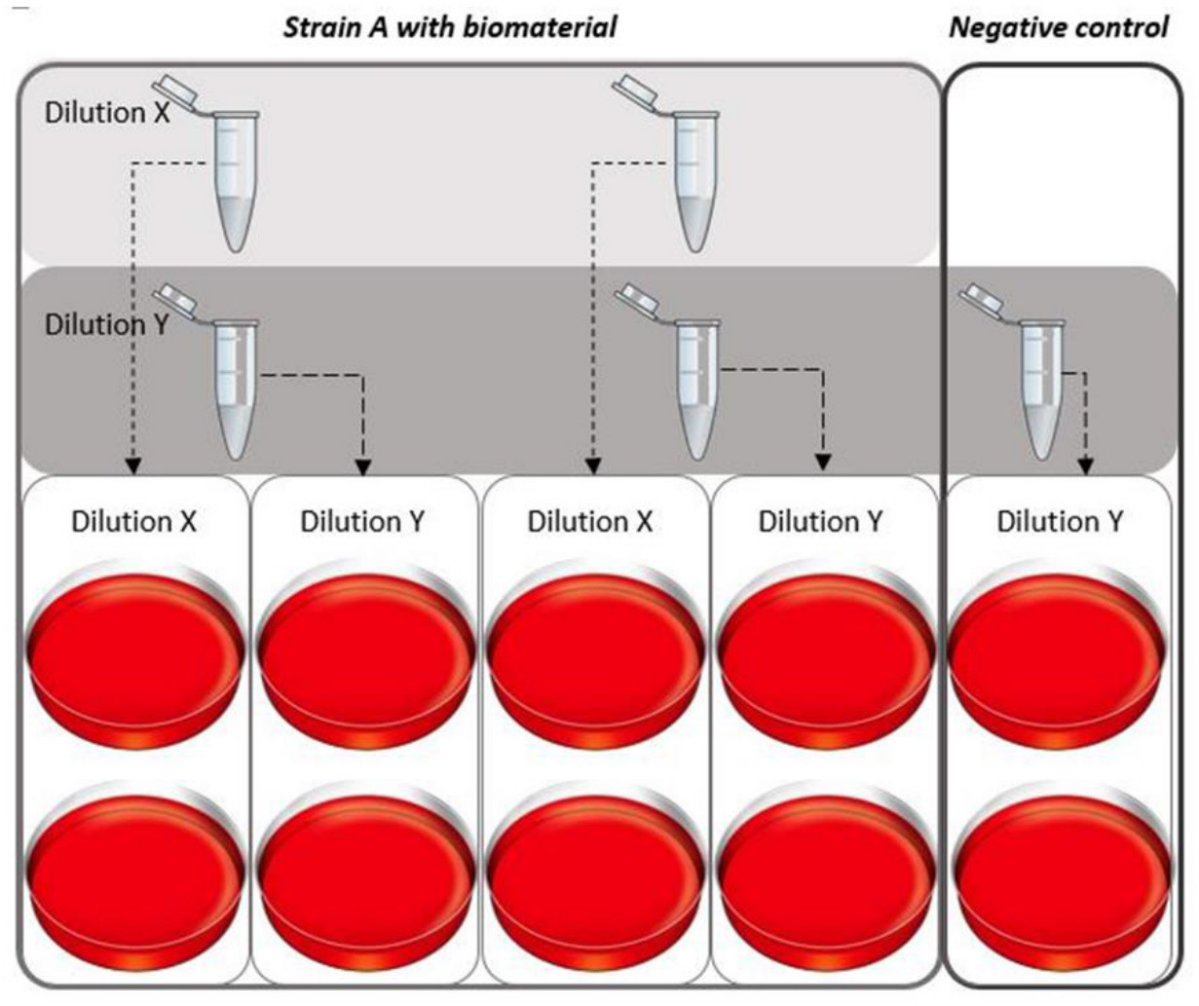
Plating dilution series of the different pathogens. For every strain a dilution series was created for each time point. Two different dilutions for the solutions with biomaterial were plated [dilution to 10^3^ CFU/mL (X) and 10^2^ CFU/mL (Y)] in duplicates. Per experiment, a growth control was cultured simultaneously, for this culture only dilution Y was plated in duplicates.

Briefly, after vortexing the complete cultured test tube, serial dilutions were prepared by mixing 100 μL from the broth with 900 μL 0.9% sodium chloride (NaCl) in water. Dilutions of 10^3^ CFU/mL (“dilution X”) and 10^2^ CFU/mL (“dilution Y”) were prepared and plated (100 μL per plate) on agar plates with 5% sheep blood (BD™ Columbia Agar with 5% Sheep Blood, Becton Dickinson GmbH, Heidelberg, Germany) in duplicates (Figure 1). For the growth control (no biomaterial, only pathogen) only dilution Y (10^2^ dilution) was plated in duplicate (Figure 1). The plates were placed on a shaking plate (IKA® KS4000 IC, IKA®-Werke GmbH & Co. KG Staufen, Germany) at 37°C and were cultured for 16–18 h before colonies were counted. The loss of broth for plating per time point (100 μL per test tube), was not replenished, since the loss of bacteria was expected to be negligible.

After 16–18 h, the agar plates were photographed and CFUs were counted using OpenCFU 3.9.0 for *MSSA, MRSA, E. coli* and *E. faecalis* (Geissmann, 2013). The CFU for *P. aeruginosa* were counted manually, since these colonies could not be detected by OpenCFU software due to low contrast of the colonies on the blood agar plate.

## Results

### pH Measurements

**Figure 2:**
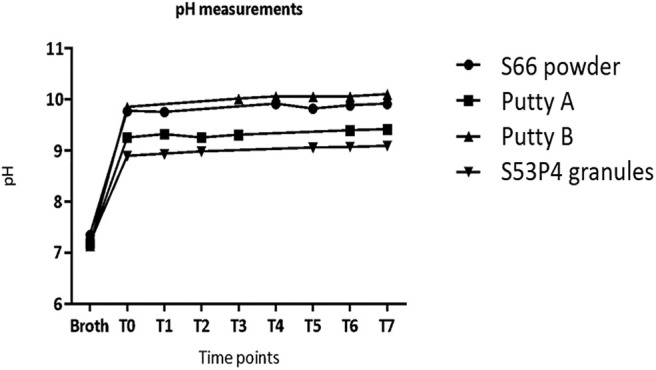
The pH values in the test tubes without the presence of pathogens increase directly after addition of material at T0 and level off in values around 9 and higher after 1 day of incubation.

The initial pH of the broths was between 7.14 and 7.35 (Figure 2). After the addition of the biomaterials, the pH increased to values of 9 and higher. The largest pH increase was observed for Putty B to 10.10 at T7 (Figure 2). The lowest pH at T7 was 9.22, observed for the S53P4 granules. After a fast increase within the first day of incubation (measured at T0), the measured pH values only slightly increased further until T7.

### Reduced CFU by the Biomaterials per Bacterial Strain

#### MSSA

**Figure 3:**
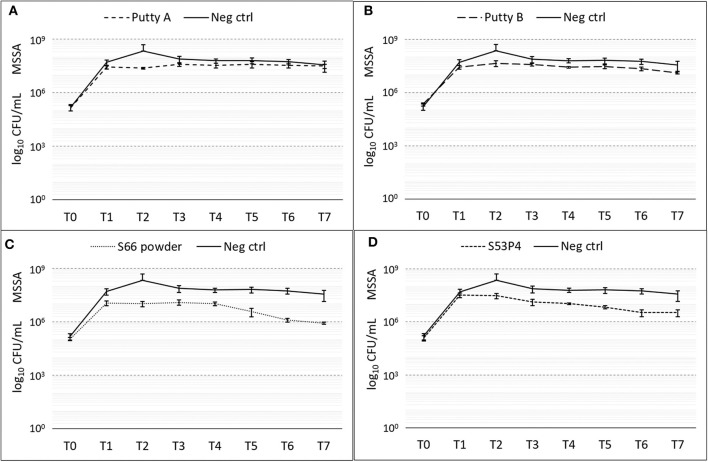
Growth of MSSA in the presence of the different biomaterials in CFU/ml compared to the growth control (continuous line in all graphs) over 7 days (T_0_ until T_7_). **(A)** shows Putty A (dashed line) compared to the growth control (continuous line), **(B)** Putty B, **(C)** S66 powder, and **(D)** SS3P4 powder. The results are presented as mean ± standard deviation on a logarithmic scale.

The number of colonies of MSSA was slightly reduced by all biomaterials (Figure 3). With a 2 log_10_ reduction of CFUs measured compared to the number measured in the growth controls, the S66 powder showed the highest reduction. S53P4 granules reduced the number of CFUs by 1 log_10_, compared to the growth control.

#### MRSA

**Figure 4:**
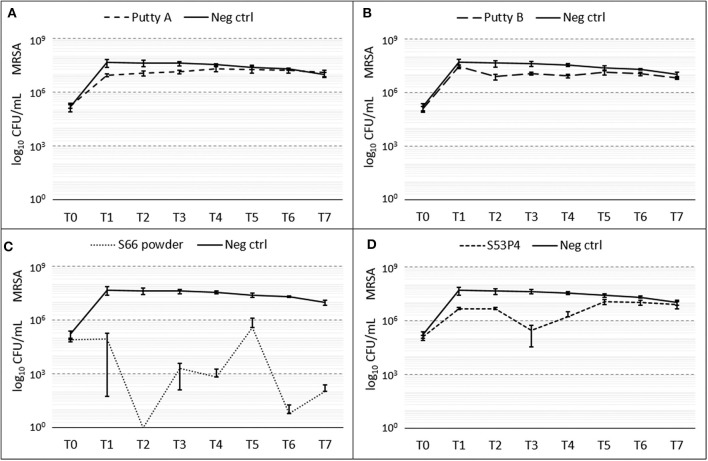
Growth of MRSA in the presence of the different biomaterials in CFU/ml compared to the growth control (continuous line in all graphs) over 7 days (T_0_ until T_7_). **(A)** shows Putty A (dashed line) compared to the growth control (continuous line), **(B)** Putty B, **(C)** S66 powder, and **(D)** SS3P4 powder. The results are presented as mean ± standard deviation on a logarithmic scale.

A slight reduction of CFUs compared to the growth control was initially observed for al biomaterials (Figure 4). Fluctuations of counted CFUs are observed for the S66 powder and the S53P4 granules. For the S66 powder, at T2 no CFUs were counted but that value of CFUs increased again. At T7, S66 powder showed a 5 log_10_ reduction in CFUs compared to the number counted in the growth control. In the S53P4 granules group, a 2 log_10_ reduction was observed compared to the growth control at T3. However, after this time point the number of counted CFUs for the S53P4 granule group increased again and at T7 this reduction was undone.

#### E. coli

**Figure 5:**
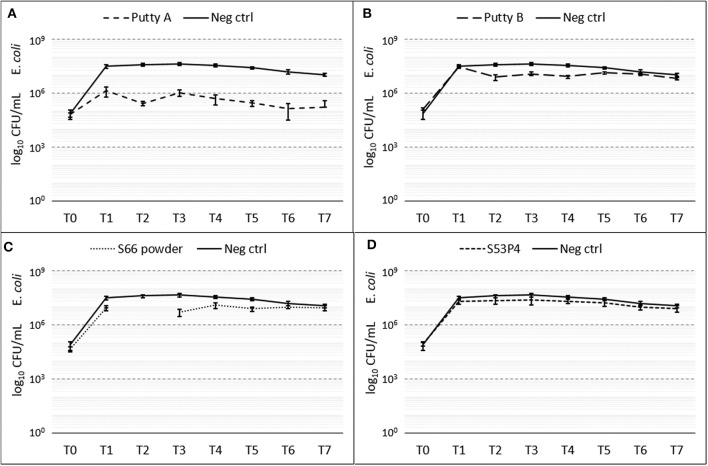
Growth of *E. coli* in the presence of the different biomaterials in CFU/ml compared to the growth control (continuous line in all graphs) over 7 days (T_0_ until T_7_). **(A)** shows Putty A (dashed line) compared to the growth control (continuous line), **(B)** Putty B, **(C)** S66 powder, and **(D)** SS3P4 powder. The results are presented as mean ± standard deviation on a logarithmic scale.

The amount of *E. coli* CFUs counted compared to the growth control were only decreased in the Putty A group with a final 2 log_10_ reduction at T7 (Figure 5). For Putty B and S66 powder a slight reduction in CFUs compared to the growth control was observed at T2 until T5, but at T7 this reduction was disappeared for both these groups. The S53P4 granules did not show any reduced counted CFUs compared to the growth control.

#### E. faecalis

**Figure 6:**
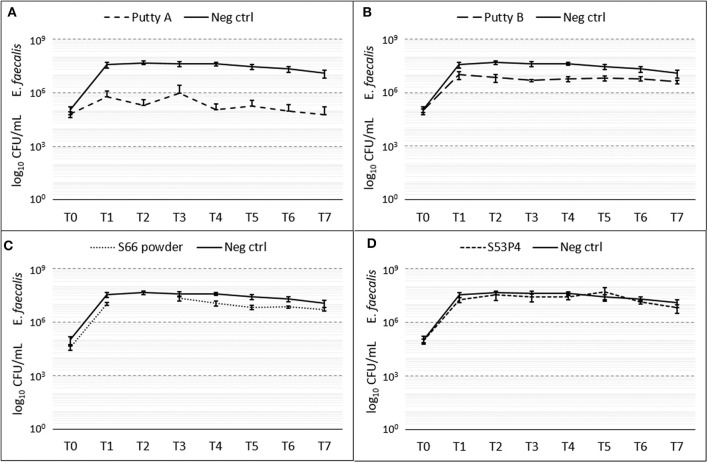
Growth of *E. faecalis* in the presence of the different biomaterials in CFU/ml compared to the growth control (continuous line in all graphs) over 7 days (T_0_ until T_7_). **(A)** shows Putty A (dashed line) compared to the growth control (continuous line), **(B)** Putty B, **(C)** S66 powder, and **(D)** SS3P4 powder. The results are presented as mean ± standard deviation on a logarithmic scale and at T2 data for the S66 powder is missing due to a contamination on the agar plate.

Putty A was most effective in eradicating *E. faecalis* showing a 2 log_10_ reduction at T_7_ (Figure 6), while the S53P4 granules and S66 powder showed almost no change in CFU compared to the growth control over time. Putty B reduced the number of counted CFUs with 1 log_10_ compared to the growth control. At T2 the data for the S66 powder are missing due to contamination on the agar plate.

#### P. aeruginosa

**Figure 7:**
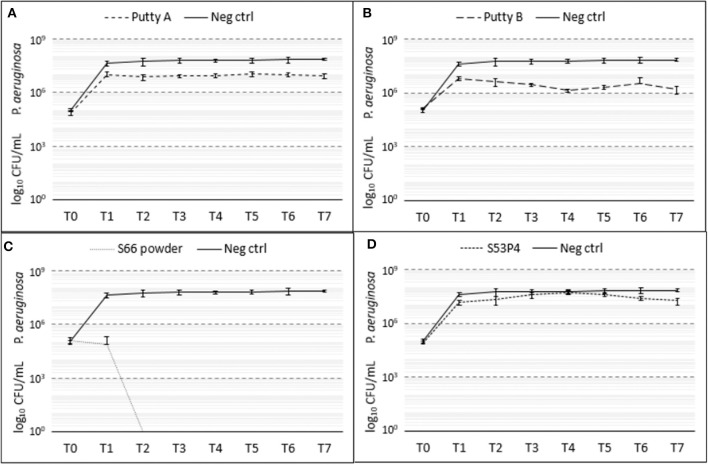
Growth of *P. aeruginosa* in the presence of the different biomaterials in CFU/ml compared to the growth control (continuous line in all graphs) over 7 days (T_0_ until T_7_). **(A)** shows Putty A (dashed line) compared to the growth control (continuous line), **(B)** Putty B, **(C)** S66 powder, and **(D)** SS3P4 powder. The results are presented as mean ± standard deviation on a logarithmic scale.

Of the four biomaterials tested, the S66 powder was the most effective in the eradication of *P. aeruginosa* as it fully eradicated the pathogen (Figure 7). Already at T1 a 3 log_10_ reduction was observed with this biomaterial, compared to the growth control. From T2 onwards, no CFUs could be detected anymore. Both putty formulations showed a reduction in the number of CFUs compared to the growth control samples. Putty A reduced the number of counted CFUs by 1 log_10_ and the Putty B 2 log_10_, at T7. The S53P4 granules did not reduce the number of CFUs compared to the growth control.

## Discussion

The goal of the current study was to screen two newly developed injectable S53P4 putty formulations on their antibacterial properties against 5 clinically relevant bacterial strains, *in vitro*. These putty formulations, consisting of S53P4 bioactive glass granules surrounded by a synthetic binder of poly(ethylene) glycol (PEG) and glycerol, were developed to improve the handling of S53P4 BAG granules. The specific composition of the synthetic binder is confidential. The study by Stoor and Frantzen (2017) observed no clear antibacterial effect for the PEG-glycerol polymer, which insinuates the antimicrobial properties of S53P4 are not enhanced by the binder (Stoor and Frantzen, 2017). The mechanical behavior of five different putty formulations is described by van Gestel et al. (2017), which showed a higher content of synthetic binder is related to increased residual strains and decreased impactability, and limited load bearing due to dissolving of the matrix (van Gestel et al., 2017).

The effective antibacterial properties of the loose S53P4 BAG granules by increasing pH and osmotic pressure have been reported previously, but it remains unclear if the incorporation of a synthetic binder, to create the putty, affect this antibacterial behavior (Leppäranta et al., 2008; Munukka et al., 2008; Zhang et al., 2010). The results obtained in this study stretch the importance that a better understanding of the underlying antimicrobial mechanism of S53P4 BAG is needed and in particular how this is related to the dosage, changes in biomaterial formulation and by changes in surface area.

Differences in capacity to reduce the number of CFUs were observed between all biomaterials and between all pathogens tested. But only in one case a full eradication of the pathogen was observed. Surprisingly, it was the S66 powder that managed to fully eradicate *P. aeruginosa*, all other biomaterials failed to completely eradicate the pathogens. Some log10 reductions in CFU/mL have been observed, but they were dependent on the biomaterial and the tested pathogen. The *in vitro* growth reduction is defined as bacteriostatic or bactericidal (Pankey and Sabath, 2004). When a material is bacteriostatic it prevents the growth of bacteria. When a material is bactericidal it means it kills the bacterial (full eradication) (Pankey and Sabath, 2004). A bacteriostatic material often shows a small growth reduction in the first 18–24 h in *in vitro* tests, while a bactericidal material reduces more than 3 log10 CFU/mL (Pankey and Sabath, 2004). Therefore, all tested biomaterials in this study showed some bacteriostatic effects. Only the S66 showed some bactericidal effects. It has been shown that S53P4 glass eradicates bacteria by a local increase in pH and osmotic pressure, created by the ions that are released upon fluid contact (Stoor et al., 1998; Munukka et al., 2008; Drago et al., 2015; van Gestel et al., 2015). As expected, the pH of the MH-II broth increased in all groups containing bioactive glass and the increase in pH was not hindered by the addition of the binder. However, the supposedly negative control, the S66 powder, even showed the highest pH increase, while the S53P4 granules showed the lowest pH increase. The relatively low pH for the S53P4 granules may be due to the used particle size of 500–80 μm, compared to the other tested biomaterials which contained particles with a size of <45 μm. It has been reported that in *in situ* measurements, the pH may differ for different particle sizes for S53P4 BAG granules in simulated body fluid (Zhang et al., 2008). The particle sizes are directly affecting the available surface area per weight of biomaterial from which ions are being released. Smaller particles have a larger surface area, which results in more ions that can be released (Stoor et al., 1998). This might also explain the high pH observed for the S66 powder, which had a much smaller particle size (<45 μm) than the S53P4 granules. A particle size of 500 μm has been related to a surface area that is 8x smaller compared to particles <45 μm (Zhang et al., 2008). Unfortunately, particle size and therefore surface area has not been homogenized in the current study and also the osmotic pressure has not been evaluated. It remains unclear from our results what caused the differences in pH for different formulations.

Two putty formulations based on the S543P4 bioactive glass were tested in this study to screen for their potential to be used in infection treatment. Our screening against five clinically relevant bacterial strains showed no complete eradication of pathogens by the new biomaterials, but neither for the one that has showed eradication of over 40 bacteria in previous tests (Leppäranta et al., 2008; Munukka et al., 2008; Zhang et al., 2010). The tests showed different effects per strain. This could be explained on a microbiological perspective since cell wall components and mucus layer are variable per species and may also may per strain. By this, the formulation and dimension of bioglass particles van vary in the antimicrobial activity in different strains and species. The difference in eradication between the two putty formulations may be based on the difference in concentration S53P4 bioactive glass. However, the putty with higher concentration S53P4 bioactive glass (Putty B) did not result in a higher eradication rate compared to Putty A, while Putty B resulted in a higher increase in pH compared to Putty A.

The supposedly negative control biomaterial, the S66 powder, showed a reduction of CFUs for several of the tested pathogens. *P. aeruginosa* was completely eradicated by this biomaterial and MSSA and MRSA showed a 2 log_10_ reduction and 5 log_10_ reduction, respectively, compared to the growth control without biomaterials. These results were unexpected and should be further investigated and understood for the assessment of new antimicrobial biomaterials in the future.

The results obtained for the control group with S53P4 granules contradict findings reported in literature, as these granules have been observed to effectively eradicate these tested pathogens (Munukka et al., 2008; Zhang et al., 2010; Drago et al., 2013). However, two of these studies did not report the antibacterial properties as a CFU reduction, but in a rather quantitatively manner. Zhang et al. (2010), reported the antimicrobial properties of S53P4 (granules with diameters smaller than 45 μm) in a classification system, with good, moderate, weak, very weak or no growth of bacteria as classifiers. No growth of *E. coli, E. faecalis*, and *P. aeruginosa* with the S53P4 powder was reported (Zhang et al., 2010). Munukka et al. (2008) reported at least a reduced growth of *S. aureus* and complete eradication of a clinical MRSA isolate and *E. coli* by S53P4 powder (granules with diameter smaller than 45 μm), based on a live-dead assay (Munukka et al., 2008). In addition, they reported a bactericidal effect on *P. aeruginosa*, which means that the growth of this pathogen was inhibited by the S53P4 powder. Whether this led to full eradication of the pathogen was not reported (Munukka et al., 2008). Not only different approaches in the quantitative vs. qualitative description of the results compared to our study could be identified. An important difference in particle size which is related to a big difference in surface area could explain the differences in results (Zhang et al., 2008). Drago et al. (2013) tested the same size of S53P4 granules as used in the current study and observed a complete eradication of *S. aureus* and *P. aeruginosa* after 72 h *in vitro* for both 400 mg glass/mL and 800 mg glass/mL broth (Drago et al., 2013). Another study used both sizes of S53P4 biomaterials (500–800 μ m granules and <45 μm powder, both 1,000 mg glass/mL MH broth) to test whether biofilms of *S. aureus*, formed on titanium discs could be effectively treated by addition of these biomaterials to the biofilm culture (Coraca-Huber et al., 2014). In their study, differences between particle sizes were observed. Both particle sizes could reduce the number of counted CFU/mL, but the powder reduced the number of colonies significantly more than the granules (Coraca-Huber et al., 2014). These results were confirmed in another recent study (Stoor and Frantzen, 2017). It needs to be determined if indeed the particle size of the S53P4 was the reason for the contradictive results, compared to literature. Additional tests with an S53P4 powder (<45 μm) should be used to validate the used approach and the currently obtained results. Full eradication of MRSA, *E. coli, E. faecalis*, and *P. aeruginosa* is expected when using the powder form (Munukka et al., 2008; Zhang et al., 2010). The surface area of the biomaterial should be standardized and the concentration of the released ions should be measured, to control the environment created by the biomaterials and define the dosage dependence. In addition, the concentration of bacteria should be taken into account. In clinical practice, the biomaterials are administered after thorough debridement of the infected area. This may result in a lower concentration of bacteria at the infection site compared to the number of bacteria used in this study. Additional research is needed to assess the influence of the mentioned variables on the antimicrobial effect.

From a clinical point of view, the larger S53P4 granules are more relevant than powder. For example in the treatment of osteomyelitis, usually bigger sized granules are used (Hulsen et al., 2017). Good clinical results have been obtained in the treatment of infections with the S53P4 granules (McAndrew et al., 2013; Geurts et al., 2016; Lindfors et al., 2016; Al Malat et al., 2018). During the treatment of osteomyelitis extensive debridement and cleaning is performed, the defect is fully packed with BAG granules, and additional systemic antibiotics are administered (Geurts et al., 2016; Lindfors et al., 2016). These aspects may contribute to the good clinical results, as potentially a much lower number of bacteria is left in the treated area after debridement compared to the used number of bacteria in the setup of this study. Furthermore, changes in osmotic pressure due to the release of ions was not measured. These aspects were not considered in our *in vitro* tests and these are therefore worst-case scenarios. A fully packed defect may result in a different concentration of ions and a locally higher pH than what was simulated in our experiments.

The current increase of antimicrobial resistance stresses the need for representative and reproducible *in vitro* tests that are predictive of the *in vivo* situation and even more the need to develop new antimicrobial biomaterials (Alanis, 2005; Kohanski et al., 2010; O'Neill, 2016; WHO, 2018). Unfortunately, the current setup did not confirm the antimicrobial activity of S53P4 BAG granules observed in patients. We could therefore not quantify to what extend the addition of a binder would affect antimicrobial properties. In addition, an inert biomaterial did show unexpected antibacterial effects, which highlights a lack of understanding of the underlying mechanisms. It has been proposed that the local increase in pH and osmotic pressure changes the morphology of bacteria and damages the cell wall (Drago et al., 2015). This mechanism would be completely different than the mechanisms reported common for antibiotics against which pathogens can develop resistance (Alanis, 2005; Kohanski et al., 2010; O'Neill, 2016; WHO, 2018). This antimicrobial resistance is a major treat for future public health and one of our biggest current challenges according the world health organization (Alanis, 2005; Kohanski et al., 2010; O'Neill, 2016; WHO, 2018). Further research is needed to evaluate the exact mechanism of S53P4 BAG and whether bacteria could develop resistance against this mechanism.

## Conclusion

This study stresses the importance of a better understanding the antimicrobial mechanism by S53P4 biomaterials and how these properties can be affected by changing the biomaterial, in size or by addition of binders to improve the handling properties. In addition, this study stresses the need to develop an *in vitro* system that is more representative of the *in vivo* situation during treatment.

## Data Availability Statement

The datasets generated for this study are available on request to the corresponding author.

## Author Contributions

ET carried out the experiments. ET and NG analyzed the data and wrote the manuscript with support from SH, JG, IL, and JA. IL, RB, and JA helped supervise the project. All authors provided critical feedback, helped shape the research, analysis, and manuscript.

## Conflict of Interest

JG and JA are clinical advisors and NG is a scientific advisor for Bonalive^®^ Biomaterials Ltd. Neither this nor the funding by Bonalive^®^ played a role in the collection, analyses, or interpretation of data; in the writing of the manuscript, or in the decision to publish the results. The remaining authors declare that the research was conducted in the absence of any commercial or financial relationships that could be construed as a potential conflict of interest.
